# Simultaneous Application of Several Exogenous dsRNAs for the Regulation of Anthocyanin Biosynthesis in *Arabidopsis thaliana*

**DOI:** 10.3390/plants13040541

**Published:** 2024-02-16

**Authors:** Konstantin V. Kiselev, Andrey R. Suprun, Olga A. Aleynova, Zlata V. Ogneva, Alexandra S. Dubrovina

**Affiliations:** Laboratory of Biotechnology, Federal Scientific Center of the East Asia Terrestrial Biodiversity, Far Eastern Branch of the Russian Academy of Sciences, 690022 Vladivostok, Russia; kiselev@biosoil.ru (K.V.K.); suprun.hi@gmail.com (A.R.S.); aleynova@biosoil.ru (O.A.A.); zlata.v.ogneva@gmail.com (Z.V.O.)

**Keywords:** dsRNA plant surface treatment, gene expression, RNAi, anthocyanin biosynthesis, dsRNA foliar application

## Abstract

Plant surface treatment with double-stranded RNAs (dsRNAs) has gained recognition as a promising method for inducing gene silencing and combating plant pathogens. However, the regulation of endogenous plant genes by external dsRNAs has not been sufficiently investigated. Also, the effect of the simultaneous application of multiple gene-specific dsRNAs has not been analyzed. The aim of this study was to exogenously target five genes in *Arabidopsis thaliana*, namely, three transcription factor genes (*AtCPC*, *AtMybL2*, *AtANAC032*), a calmodulin-binding protein gene (*AtCBP60g*), and an anthocyanidin reductase gene (*AtBAN*), which are known as negative regulators of anthocyanin accumulation. Exogenous dsRNAs encoding these genes were applied to the leaf surface of *A. thaliana* either individually or in mixtures. The mRNA levels of the five targets were analyzed using qRT-PCR, and anthocyanin content was evaluated through HPLC-MS. The results demonstrated significant downregulation of all five target genes by the exogenous dsRNAs, resulting in enhanced expression of chalcone synthase (*AtCHS*) gene and increased anthocyanin content. The simultaneous foliar application of the five dsRNAs proved to be more efficient in activating anthocyanin accumulation compared to the application of individual dsRNAs. These findings hold considerable importance in plant biotechnology and gene function studies.

## 1. Introduction

The task of developing innovative approaches to modify various plant traits, while keeping their genome intact, is of great importance in modern plant biotechnology. While transgenic plants and transgene-free genome editing [1] have proven to be effective approaches to managing plant traits, there is still a significant lack of understanding of the potential consequences of genetic modifications and genome editing in plants. This has sparked intense debates surrounding the safety of genetically modified or edited organisms, leading to numerous countries imposing legal restrictions on the production and cultivation of such organisms [2,3].

Plant surface treatment with aqueous solutions of double-stranded RNAs (dsRNAs), which is known as spray-induced gene silencing or SIGS, is an emerging strategy for manipulating plant characteristics. This innovative technique is currently being extensively researched as a viable alternative to genetically modified plants [4,5,6]. dsRNAs are a key inducer of the RNA interference (RNAi)-based gene silencing processes in eukaryotic organisms. During RNAi-mediated gene silencing, specialized enzymes known as Dicer-like or DCL, convert the dsRNAs into short interfering RNAs or siRNAs [7,8]. Subsequently, these siRNAs are integrated into the RNA-induced silencing complex (RISC). The primary function of the RISC is to degrade any RNA molecules that possess a similarity to the dsRNAs. Consequently, the mRNAs of genes that share significant homology with the dsRNA-derived siRNAs are destroyed, resulting in decreased expression of these genes.

At present, the RNAi phenomenon has become a prevalent technique utilized in experimental biology to effectively inhibit gene expression in plants. This technique is instrumental in studying gene functions as well as modifying desirable plant characteristics to meet specific needs and requirements [9,10,11]. However, the application of this approach necessarily involves the stage of obtaining a transgenic plant transformed by a dsRNA-encoding vector construct or the application of dsRNA-encoding constructs based on attenuated plant viruses for subsequent plant infection without viral infection symptoms [9,12]. 

At present, multiple studies have been conducted reporting on plant pathogen protection using the SIGS approach with the application of exogenous dsRNAs designed to inhibit the expression of virulence-associated genes in the attacking pathogens [4,11,13,14]. It has been established that fungal pathogens can efficiently internalize exogenous dsRNAs applied to the plant surface. Once internalized, these dsRNAs undergo processing and subsequently trigger the silencing of the targeted pathogen genes [13,15,16]. Furthermore, several intriguing studies have demonstrated that external dsRNAs, which are designed to target plant genes, may lead to the down-regulation of the gene targets in the plant genome with the consequent biochemical or phenotypic changes, such as altered flower morphology, reductions in glucose and fructose content, drought stress tolerance, changed anthocyanin production, or fungal stress resistance [17,18,19,20,21,22]. There have also been studies demonstrating that the suppression of targets in plants was attained through nanoparticle-mediated or laser light-guided external treatments with dsRNAs [23,24,25]. The externally applied dsRNAs have been detected in the plant cells and the plant vascular system [15,19,23]. However, the number of reports on externally induced silencing of plant targets is scarce in the current literature, and further studies are required. The discovery of the ability to regulate the level of plant gene expression by applying dsRNA to the surface of the plant opens up new possibilities for functional studies on plant genes and for regulating plant traits for the desired time in agriculture.

**Figure 1:**
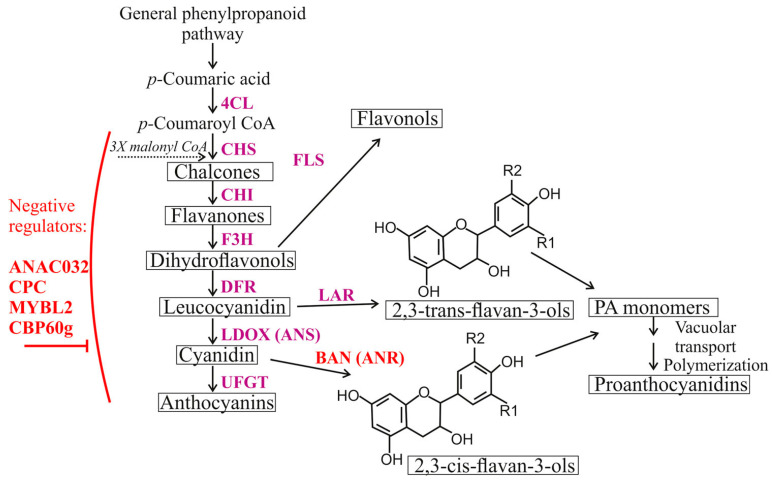
The pathway of anthocyanin biosynthesis. The 4-coumarate-CoA ligase (4CL) is the enzyme involved in general phenylpropanoid pathway. The enzymes participating in the anthocyanin biosynthesis pathway encompass chalcone synthase (CHS), chalcone isomerase (CHI), flavanone 3-hydroxylase (F3H), dihydroflavonol 4-reductase (DFR), anthocyanidin synthase (ANS or LDOX), anthocyanidin/flavonol 3-*O*-glucosyltransferase (UFGT), flavanonol synthase (FLS), leucoanthocyanidin reductase (LAR), and anthocyanidin reductase (ANR or BAN). Enzymes of each step are shown in purple. Negative regulators of the anthocyanin biosynthesis and the gene target from a competing pathway are shown in red.

Our recent work has shown that external treatment of the *Arabidopsis thaliana* leaf surface with aqueous solutions of dsRNAs and siRNAs that specifically target the chalcone synthase *AtCHS* and two transcription repressor *AtMybL2* and *AtANAC032* genes, which are implicated in anthocyanin biosynthesis and its regulation, led to a significant target down-regulation and corresponding changes in anthocyanin levels [19]. Anthocyanins, which are derived from the phenylpropanoid biosynthetic pathway (as shown in Figure 1), are a type of colored secondary metabolites. These compounds are well-known for their valuable properties that have significant applications in various fields such as medicine, the food industry, cosmetology, and ornamental horticulture [26,27]. It has been shown that exogenous *AtCHS*-dsRNA penetrates into the vascular system and individual plant cells of *A. thaliana* and spreads through the vascular system and in groups of parenchymal cells [19]. We also found that the decrease in the *AtCHS* expression levels was associated with the appearance of a fraction of *AtCHS*-specific small RNAs against this gene and was presumably induced by RNAi-mediated processes [28]. 

This investigation was aimed to increase the content of anthocyanins in *A. thaliana* by utilizing exogenous dsRNAs that specifically target five genes in *A. thaliana*, namely, three transcription factor genes (*AtCPC*, *AtMybL2*, *AtANAC032*), a calmodulin-binding protein gene (*AtCBP60g*), and an anthocyanidin reductase gene (*AtBAN*). The genes of single-repeat R3 MYB transcription factors AtCPC and AtMybL2 [29,30], a NAC transcription factor AtANAC032 [31], and a calmodulin-binding protein AtCBP60g [32] are known as negative regulators (Figure 1) of anthocyanin accumulation in *A. thaliana* [29,30,31], while the *AtBAN* (*AtANR*) gene encodes an anthocyanin reductase (Figure 1) converting anthocyanidins to 2,3-*cis*-flavan-3-ols in a competing pathway [33]. According to data in the literature, mybl2, cpc-1, cbp60g mutant plant lines and chimeric repressor line ANAC032-SRDX (phenotype similar to loss-of-function mutants) exhibited upregulated expression levels of *AtCHS* and some other anthocyanin biosynthetic genes in *A. thaliana* [29,30,31,32]. This was also accompanied by an increased content of anthocyanins. At the same time, overexpression lines were, in turn, characterized by downregulated *AtCHS*. Therefore, downregulation of these “negative regulators” should lead to increased mRNA levels of the *AtCHS* gene and anthocyanin production. To our understanding, there is no information regarding the simultaneous application of several dsRNAs in a mixture for the downregulation of gene targets in plants. We also evaluated individual effects of the five dsRNAs on target gene expression, anthocyanin content, and *AtCHS* expression.

## 2. Results 

### 2.1. Effect of Exogenous dsRNAs Encoding AtANAC032, AtBAN, AtCBP60g, AtCPC, and AtMYBL2, Applied Separately, on the Accumulation of mRNAs of These Target Genes in A. thaliana

**Figure 2:**
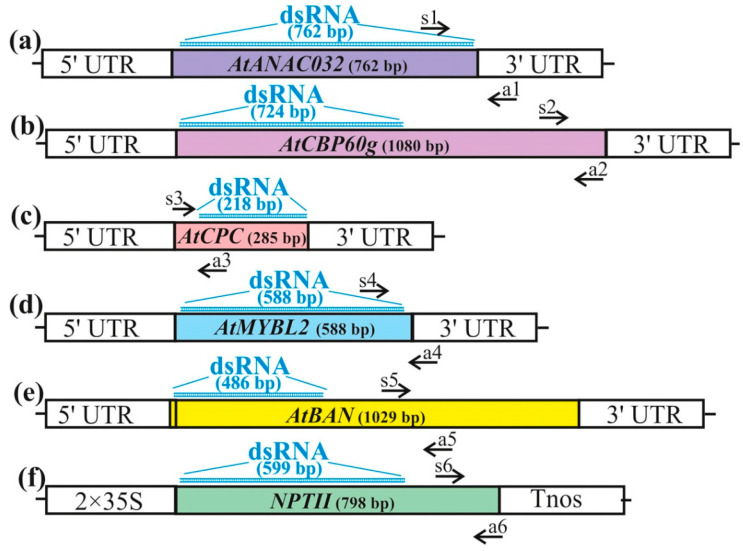
The dsRNA design and primer positions used to assess the impact of exogenous dsRNAs on the *AtANAC032, AtCBP60g*, *AtCPC*, *AtMYBL2*, and *AtBAN* gene expressions. The coding regions of each gene along with the dsRNA and primer positions are depicted as follows: (**a**) *AtANAC032* along with the dsRNA and primer positions; (**b**) *AtCBP60g* along with the sRNA and primer positions; (**c**) *AtCPC* along with the dsRNA and primer positions; (**d**) *AtMYBL2* along with the dsRNA and primer positions; (**e**) *AtBAN* along with the dsRNA and primer positions; (**f**) *NPTII* along with the dsRNA and primer positions. The black arrows show the primer pair positions (s1-a1, s2-a2, s3-a3, s4-a4, s5-a5, s6-a6) employed for the analysis of the mRNA levels of the *AtANAC032*, *AtCBP60g*, *AtCPC*, *AtMYBL2*, and *AtBAN* genes. UTR—untranslated region, 2 × 35S—the double 35S promoter of the cauliflower mosaic virus (CaMV), Tnos—nopaline synthase terminator.

In order to assess the effectiveness of five external dsRNAs in inhibiting gene expression in *A. thaliana*, five genes of *AtANAC032, AtCBP60g*, *AtCPC*, and *AtMYBL2*, and *AtBAN* were selected. Five gene-specific dsRNAs of 762, 724, 218, 588, and 486-bp in length, respectively, were synthesized using PCR and an in vitro transcription protocol based on the *AtANAC032, AtCBP60g*, *AtCPC*, *AtMYBL2*, and *AtBAN* gene sequences of *A. thaliana* for the exogenous RNAi experiments (Figure 2). In order to confirm the specificity of the observed effects induced by dsRNAs, we synthesized dsRNA targeting an unrelated gene of a bacterial neomycin phosphotransferase II *NPTII* (Figure 2). The full-length cDNAs of *AtANAC032*, *AtCPC*, and *AtMYBL2*, and large cDNA fragments of *AtCBP60g* and *AtBAN*, were amplified using primers that contained the T7 RNA polymerase promoter (Figure 2; Table S1). 

PCR products were subjected to modification by incorporating T7 promoters at both ends, facilitating their utilization as templates during the in vitro transcription process. To treat external plant surfaces, a solution containing 35 µg of a dsRNA was prepared by diluting it in 100 µL of water (0.35 µg/µL). This solution was then gently spread onto the leaves of each individual *A. thaliana* plant using sterile soft brushes [19,34]. According to our previous analyses [19,34,35], our observations showed that considerable gene silencing of a transgene in *A. thaliana* can be achieved by treating four-week-old rosettes during the late hours of the day (21:00–21:30) under conditions of low soil moisture conditions. Thus, we decided to focus on these parameters during the current experiments. 

**Figure 3:**
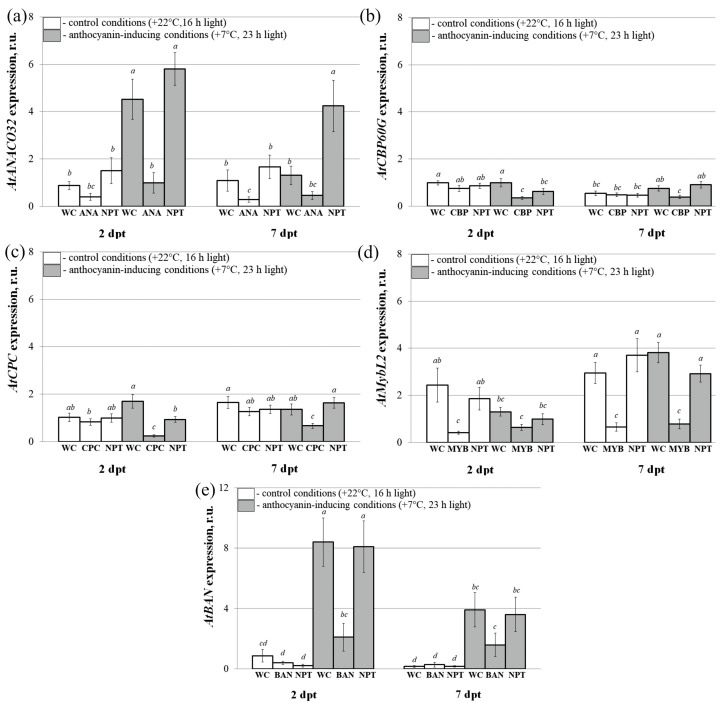
The relative fold change in the mRNA levels of *AtANAC032* (**a**), *AtCBP60g* (**b**), *AtCPC* (**c**), *AtMYBL2* (**d**), and *AtBAN* (**e**) after treatment with individual dsRNAs in *Arabidopsis thaliana* compared to untreated plants. The treatment groups included *A. thaliana* treated with sterile water (WC), *ANAC032*-dsRNA (ANA), *AtCBP60g*-dsRNA (CBP), *AtCPC*-dsRNAs (CPC), *AtMYBL2*-dsRNAs (MYB), *AtBAN*-dsRNAs (BAN), and *NPTII*-dsRNA (NPT). Total RNA was extracted at two and seven days following the application of dsRNA, with subsequent gene expression analysis performed using qRT-PCR. The treated *A. thaliana* rosettes were separated into two groups for incubation. The first group was exposed to control conditions (+22 °C, 16 h light), while the second group to anthocyanin-inducing conditions (+7 °C, 23 h light). The data are presented as the mean ± SE (three independent experiments). Means in each figure followed by the same letter were not different when Student’s *t* test was used (*p* < 0.05).

We then investigated the effect of the individual exogenous *AtANAC032*, *AtCBP60g*, *AtCPC*, *AtMYBL2*, and *AtBAN* applied separately on the accumulation of mRNAs of the target genes compared to the control water and *NPTII*-dsRNAs treatments (Figure 3). Due to the insufficient anthocyanin levels and limited activity of anthocyanin biosynthesis genes observed under normal growth conditions, the treated *A. thaliana* rosettes were categorized into two groups and subjected to post-treatment incubation. The first group was exposed to control conditions (+22 °C, 16 h light), while the second group was exposed to anthocyanin-inducing conditions (+7 °C, 23 h light) for two and seven days. This differential treatment aimed to stimulate anthocyanin biosynthesis for further analysis.

qRT-PCR was utilized to examine the expression of the five target genes (Figure 3). According to the analysis, the expression levels of the *AtANAC032*, *AtCBP60g*, *AtCPC*, *AtMYBL2*, and *AtBAN* target genes were lower after foliar treatments with the five individual dsRNAs compared to the water or the non-specific *NPTII*-dsRNA treatments. Spreading the *AtANAC032*, *AtCBP60g*, *AtCPC*, *AtMYBL2*, and *AtBAN* dsRNAs under the conditions of reduced temperature and prolonged illumination lowered the expression of the target genes by 2–5.9-fold. It is important to note that the external application of the non-specific *NPTII*-dsRNA to plants did not have a substantial impact on the expression levels of *AtANAC032*, *AtCBP60g*, *AtCPC*, *AtMYBL2*, and *AtBAN* genes when compared to the control group treated with water. This finding confirms that the gene silencing effects induced by dsRNA were sequence-specific.

### 2.2. Simultaneous Application of Five dsRNAs Encoding the Genes AtANAC032, AtCBP60g, AtCPC, AtMYBL2, and AtBAN in Mixtures for the Target Gene Silencing in A. thaliana

To analyze the effect of the five dsRNAs applied in combination externally for the regulation of the *AtANAC032*, *AtCBP60g*, *AtCPC*, *AtMYBL2*, and *AtBAN* genes, we prepared mixtures of the five gene-specific dsRNAs containing 10, 20, or 30 µg of each dsRNA, i.e., 50 µg, 100 µg, or 150 µg of the dsRNAs mixed together (in 100 µL of water per individual plant). The leaf surface of four-week-old *A. thaliana* rosettes was treated externally with three different dsRNA mixtures. The mixtures of five dsRNAs targeting the *AtANAC032*, *AtCBP60g*, *AtCPC*, *AtMYBL2*, and *AtBAN* genes were applied to the leaf surface of four-week-old *A. thaliana* rosettes, both on the upper (adaxial) and lower (abaxial) sides, following the procedure described above for the individual application of the five dsRNAs.

**Figure 4:**
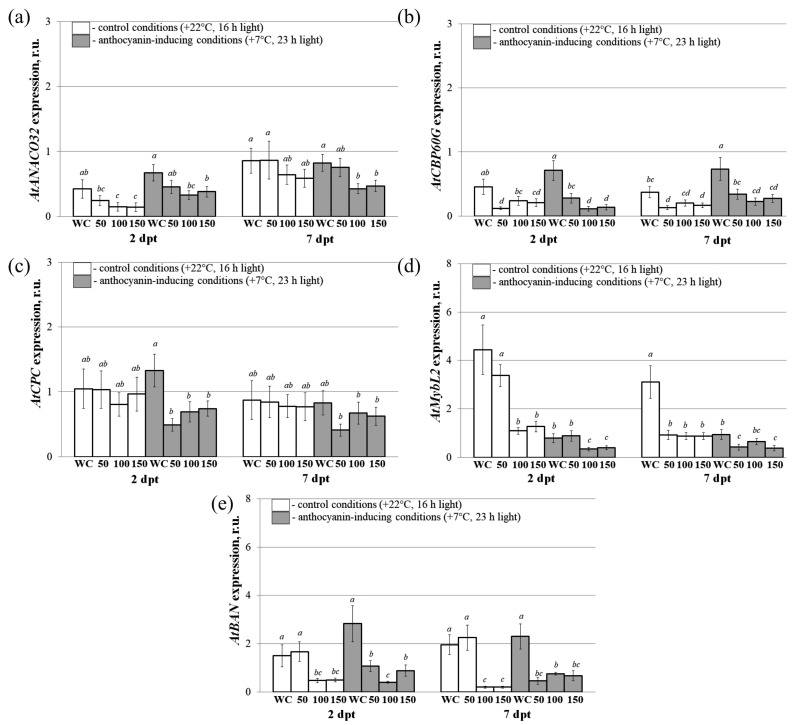
The relative fold change in the mRNA levels of *AtANAC032* (**a**), *AtCBP60g* (**b**), *AtCPC* (**c**), *AtMYBL2* (**d**), and *AtBAN* (**e**) after treatment with dsRNA mixtures in *Arabidopsis thaliana* compared to untreated plants. The dsRNA mixtures used were as follows: 50—mixture of the five gene-specific *dsRNAs* containing 10 µg of each dsRNA (*AtANAC032*, *AtCBP60g*, *AtCPC*, *AtMYBL2*, and *AtBAN*-specific dsRNAs); 100—mixture of the five gene-specific dsRNAs containing 20 µg of each dsRNA; 150—mixture of the five gene-specific dsRNAs containing 30 µg of each dsRNA; WC—plants treated with sterile water. Total RNA was extracted at two and seven days following the application of dsRNA, with subsequent gene expression analysis performed using qRT-PCR. The treated *A. thaliana* rosettes were separated into two groups for incubation. The first group was exposed to control conditions (+22 °C, 16 h light), while the second group to anthocyanin-inducing conditions (+7 °C, 23 h light). The data are presented as the mean ± SE (three independent experiments). Means in each figure followed by the same letter were not different when the Student’s *t* test was used (*p* < 0.05).

We then investigated the effect of the exogeneous application of the multiple dsRNAs in mixtures on the expression of the five target genes in *A. thaliana* compared to the control group treated with water or *NPTII*-dsRNA two and seven days after treatment (Figure 4). For this purpose, the treated *A. thaliana* rosettes were separated into two groups for incubation. The first group was kept under control conditions, with a temperature of +22 °C and 16 h of light. The second group was exposed to conditions that induce anthocyanin production, with a temperature of +7 °C and 23 h of light.

Exogenous application of 100 and 150 µg dsRNA mixtures resulted in a marked decrease in the transcript level of *AtANAC032* (Figure 4a). This down-regulation effect was observed to be statistically significant at both two and seven days after treatment under the conditions of modulating anthocyanin biosynthesis. However, under control conditions, the down-regulation effect was only observed at two days post-treatment (Figure 4a). *AtCBP60G* and *AtBAN* expression exhibited a significant decrease after two and seven days of treatment with dsRNA under anthocyanin-inducing conditions for all types of mixtures (Figure 4b,e). We also observed significantly reduced *AtCBP60G* and *AtBAN* transcript levels for control conditions, and the effect was statistically significant for 50 and 150 µg for *AtCBP60G* and for 100 and 150 µg for *AtBAN*. *AtCPC* expression was considerably downregulated two days after treatment only under the anthocyanin-inducing conditions for all types of mixtures and for 50 µg seven days after treatment (Figure 4c). Similar to *AtANAC032*, mRNA levels of *AtMYBL2* were markedly downregulated two days after the application of 100 and 150 µg dsRNA mixtures under both control and anthocyanin-inducing conditions compared to the water-treated control, while the down-regulation effect of 50 µg dsRNA mixture was not statistically significant (Figure 4d). We also observed a similar effect of a decrease in the expression of this gene seven days after the application of all types of mixtures under control conditions and for 50 µg and 150 µg under anthocyanin-inducing conditions.

### 2.3. The Effect of Individual dsRNAs on the AtCHS mRNA Levels and Anthocyanin Content in A. thaliana

**Figure 5:**
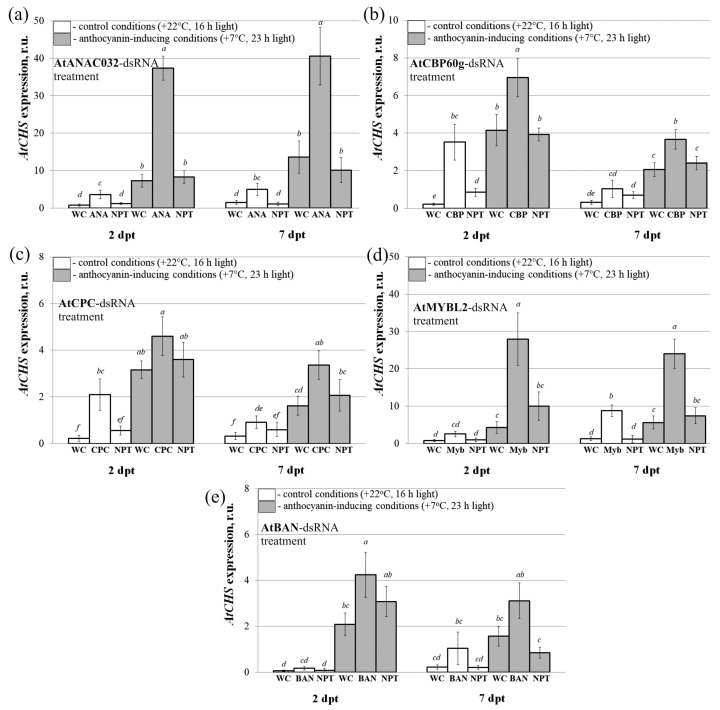
The relative fold change in *AtCHS* mRNA level after treatment of *Arabidopsis thaliana* with individual dsRNAs encoding the *AtANAC032* (**a**), *AtCBP60g* (**b**), *AtCPC* (**c**), *AtMYBL2* (**d**), *AtBAN* (**e**), and *NPTII* genes compared to untreated plants. The treatment groups included *A. thaliana* treated with sterile water (WC), *ANAC032*-dsRNA (ANA), *AtCBP60g*-dsRNA (CBP), *AtCPC*-dsRNAs (CPC), *AtMYBL2*-dsRNAs (MYB), *AtBAN*-dsRNAs (BAN), and *NPTII*-dsRNA (NPT). Total RNA was extracted at two and seven days following the application of dsRNA, with subsequent gene expression analysis performed using qRT-PCR. The treated *A. thaliana* rosettes were separated into two groups for incubation. The first group was exposed to control conditions (+22 °C, 16 h light), while the second group to anthocyanin-inducing conditions (+7 °C, 23 h light). The data are presented as the mean ± SE (three independent experiments). Means in each figure followed by the same letter were not different using Student’s *t* test (*p* < 0.05).

**Figure 6:**
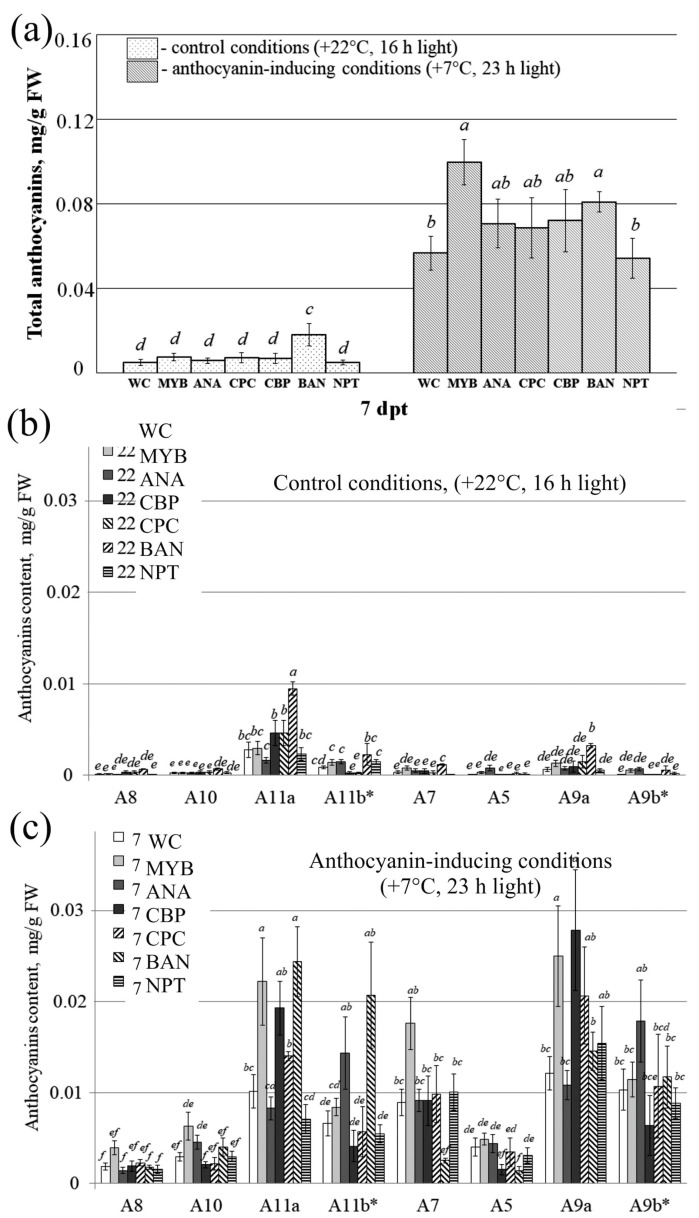
Anthocyanin content in the leaves of *Arabidopsis thaliana* seven days after treatment with the individual dsRNAs. (**a**) The total anthocyanin content; (**b**) the content of individual anthocyanins under control conditions; (**c**) the content of individual anthocyanins under the anthocyanin modulatory conditions. The treatment groups included *A. thaliana* treated with sterile water (WC), *ANAC032*-dsRNA (ANA), *AtCBP60g*-dsRNA (CBP), *AtCPC*-dsRNAs (CPC), *AtMYBL2*-dsRNAs (MYB), *AtBAN*-dsRNAs (BAN), and *NPTII*-dsRNA (NPT). The data are presented as the mean ± SE (three independent experiments). Means in each figure followed by the same letter were not different when using Student’s *t* test (*p* < 0.05). A8—Cyanidin 3-*O*-[2″-*O*-(xylosyl) 6″-*O*-(*p*-O-(glucosyl) *p*-coumaroyl) glucoside] 5-*O*-[6‴-*O*-(malonyl) glucoside]; A10—Cyanidin 3-*O*-[2″-*O*-(2‴-*O*-(sinapoyl) xylosyl) 6″-*O*-(*p*-*O*-(glucosyl) *p*-coumaroyl) glucoside] 5-*O*-glucoside; A11a, A11b*—Cyanidin 3-*O*-[2″-*O*-(6‴-*O*-(sinapoyl) xylosyl) 6″-*O*-(*p*-*O*-(glucosyl)-*p*-coumaroyl) glucoside] 5-*O*-(6′′′′-*O*-malonyl) glucoside; A7—Cyanidin 3-*O*-[2″-*O*-(2‴-*O*-(sinapoyl) xylosyl) 6″-*O*-(*p*-coumaroyl) glucoside] 5-*O*-glucoside; A5—Cyanidin 3-*O*-[2″-*O*-(xylosyl)-6″-*O*-(*p*-coumaroyl) glucoside] 5-*O*-malonylglucoside; A9a, A9b*—Cyanidin 3-*O*-[2″-*O*-(2‴-*O*-(sinapoyl) xylosyl) 6″-*O*-(*p*-*O*-coumaroyl) glucoside] 5-*O*-[6′′′′-*O*-(malonyl) glucoside]. * Asterisk indicates a tautomer. The names of the anthocyanins are presented in accordance with previously published data [35].

Using qRT-PCR, we also analyzed the effect of exogenous *AtANAC032*, *AtBAN*, *AtCBP60g*, *AtCPC*, and *AtMYBL2*-coding dsRNAs (separately) on the transcript levels of *AtCHS*, which encodes an important enzyme involved in anthocyanin biosynthesis (Figure 1). The anthocyanin content was analyzed using the HPLC with a high-resolution mass spectrometry (HPLC-MS) analysis (Figure 5 and Figure 6).

The results demonstrated that under anthocyanin-inducing conditions, the cultivation of *A. thaliana* resulted in a notable increase in *AtCHS* transcript levels compared to the control conditions (Figure 5a–e). This indicated the successful activation of anthocyanin biosynthesis under the applied conditions. Specifically, we observed a 1.1–16.5-fold increase in *AtCHS* mRNA levels two days after treatment, and a 1.1–6.7-fold increase seven days after treatment following the application of the gene-specific dsRNAs. 

The HPLC analysis of total anthocyanin profile revealed that plants treated with *AtMYBL2* and *AtBAN*-dsRNAs displayed a significantly higher total anthocyanin content compared to the control group treated with either water or *NPTII*-dsRNA (Figure 6a). The anthocyanin content in the dsRNA-treated plants reached 0.08–0.10 mg/g FW under anthocyanin-inducing conditions. While treatment with *AtANAC032*, *AtCBP60g*, and *AtCPC*-dsRNAs also resulted in increased total anthocyanin content (up to 0.07 mg/g FW), the increase was not statistically significant. The application of *AtMYBL2*-dsRNA resulted in the highest total anthocyanin level of 0.10 mg/g FW and was associated with a pronounced upregulation of the *AtCHS1* gene expression (Figure 5d and Figure 6a). *AtBAN*-dsRNA modulated total anthocyanins both in the control and anthocyanin-modulated groups (Figure 6a). 

Eight individual anthocyanin compounds were identified through the HPLC-MS analysis in the externally treated leaves of *A. thaliana* of all groups both under control conditions (Figure 6b and Figure S1, Table S2) and conditions where anthocyanin levels were modulated (Figure 6c and Figure S1, Table S2). Compared to the control conditions, we found that the plants grown under the high-light and cold-stress conditions exhibited higher levels of most individual anthocyanins (Figure 6b,c). Plant treatments with exogenous *AtMYBL2*, *AtANAC032*, *AtCBP*, and *AtBAN*-dsRNAs significantly enhanced the levels of several individual anthocyanins, with these changes being statistically significant for A11a and A9a (Figure 6b,c). It is important to note that the control non-specific dsRNA on *NPTII* did not affect the total level of anthocyanins (Figure 6a), the variety of anthocyanin compounds (Figure 6b,c), or *AtCHS* transcript levels (Figure 5). The findings provide further evidence of the targeted gene silencing effects mediated by gene-specific dsRNA.

### 2.4. The Effect of the Five dsRNAs Applied in Mixtures on the AtCHS mRNA and Anthocyanin Levels in A. thaliana

**Figure 7:**
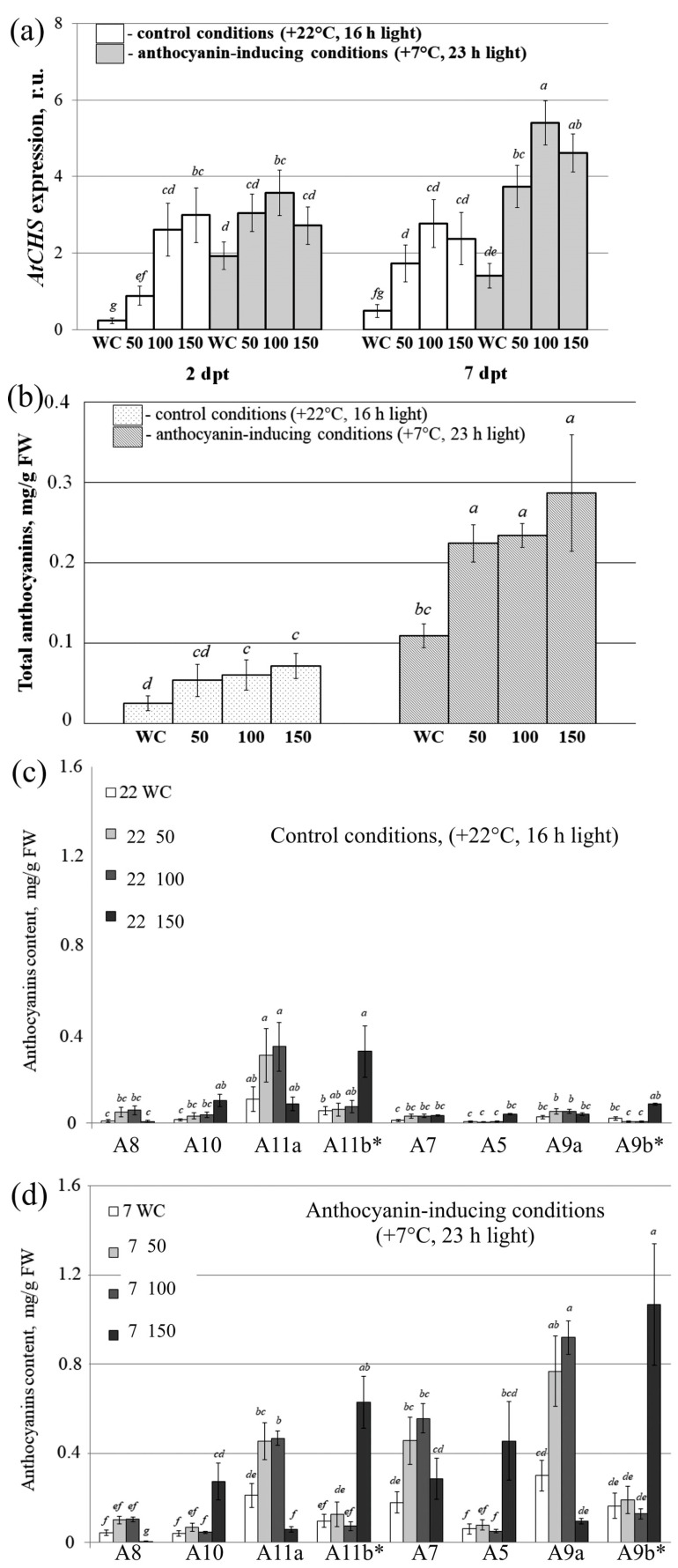
*AtCHS* transcript and anthocyanin levels in *Arabidopsis thaliana* leaves examined seven days after treatment with dsRNA mixtures of five dsRNAs of *AtANAC032*, *AtCBP60g*, *AtCPC*, *AtMYBL2*, and *AtBAN* genes. (**a**) Relative fold change in *AtCHS* mRNA level after treatment of *A. thaliana* with dsRNAs mixtures; (**b**) the total anthocyanin content; (**c**) the content of individual anthocyanins under control conditions; (**d**) the content of individual anthocyanins under anthocyanin-inducing conditions. The dsRNA mixtures used were as follows: 50—mixture of the five gene-specific dsRNAs containing 10 µg of each dsRNA; 100—mixture of the five gene-specific dsRNAs containing 20 µg of each dsRNA; 150—mixture of the five gene-specific dsRNAs containing 30 µg of each dsRNA. Data are presented as the mean ± SE (three independent experiments). Means in each figure followed by the same letter were not different when using Student’s *t* test (*p* < 0.05). A8—Cyanidin 3-*O*-[2″-*O*-(xylosyl) 6″-*O*-(*p*-O-(glucosyl) *p*-coumaroyl) glucoside] 5-*O*-[6‴-*O*-(malonyl) glucoside]; A10—Cyanidin 3-*O*-[2″-*O*-(2‴-*O*-(sinapoyl) xylosyl) 6″-*O*-(*p*-*O*-(glucosyl) *p*-coumaroyl) glucoside] 5-*O*-glucoside; A11a, A11b*—Cyanidin 3-*O*-[2″-*O*-(6‴-*O*-(sinapoyl) xylosyl) 6″-*O*-(*p*-*O*-(glucosyl)-*p*-coumaroyl) glucoside] 5-*O*-(6′′′′-*O*-malonyl) glucoside; A7—Cyanidin 3-*O*-[2″-*O*-(2‴-*O*-(sinapoyl) xylosyl) 6″-*O*-(*p*-coumaroyl) glucoside] 5-*O*-glucoside; A5—Cyanidin 3-*O*-[2″-*O*-(xylosyl)-6″-*O*-(*p*-coumaroyl) glucoside] 5-*O*-malonylglucoside; A9a, A9b*—Cyanidin 3-*O*-[2″-*O*-(2‴-*O*-(sinapoyl) xylosyl) 6″-*O*-(*p*-*O*-coumaroyl) glucoside] 5-*O*-[6′′′′-*O*-(malonyl) glucoside]. * Asterisk indicates a tautomer. The names of the anthocyanins are presented in accordance with previously published data [35].

We then analyzed the influence of exogenous *AtANAC032*, *AtBAN*, *AtCBP60g*, *AtCPC*, and *AtMYBL2*-encoding dsRNAs applied simultaneously in mixtures on the expression of the *AtCHS* gene through qRT-PCR (Figure 7a) and anthocyanin content through HPLC-MS analysis (Figure 7b–d).

The expression of *AtCHS* and anthocyanin content were significantly increased in the control WC plants under the anthocyanin modulatory conditions, indicating that anthocyanin biosynthesis was successfully induced (Figure 7a,b). We found that the *AtCHS* transcript levels were increased 1.9–13.0 times two days post treatment and 3.8–5.7 times seven days post treatment after the application of the gene-specific dsRNA for both anthocyanin-inducing and control conditions (Figure 7a).

The HPLC analysis of the anthocyanin profile showed that *A. thaliana* treated with all three types of dsRNA mixtures (i.e., 50, 100, and 150 µg) contained significantly higher total amounts of anthocyanins, reaching 0.06–0.29 mg/g FW, than the plants treated with water under both control and anthocyanin-inducing conditions, except for 50 µg under control conditions (Figure 7b). Utilizing high doses of dsRNA under anthocyanin-inducing conditions resulted in the most notable accumulation of total anthocyanins at 0.29 mg/g FW, accompanied by the highest increase in expression of the *AtCHS* gene (Figure 7b). 

HPLC-MS analysis demonstrated that leaves of *A. thaliana* treated with water and dsRNA contained identical eight anthocyanin compounds as observed under the control conditions (Figure 7c) and anthocyanin modulation conditions (Figure 7d). Plant treatment with the three dsRNA mixtures resulted in considerable elevation in the levels of most individual anthocyanins under anthocyanin-inducing condition, except for A8 (Figure 7d). Under control conditions, we observed less pronounced elevations in the content of anthocyanins with considerable changes in the content of A10, A11a, and A11b.

## 3. Discussion 

In modern society, there is a need to develop new effective approaches to protect and increase plant productivity using safe and environmentally friendly technologies. The increasing human population and the adverse effects of environmental stresses are driving the need for new molecular tools to improve and protect crops without altering the plant genome. Recently, a new approach, SIGS, has gained popularity for altering plant characteristics in the desired direction [4,5,36,37]. This approach includes treating plant surfaces with dsRNA solutions and induces RNAi-mediated silencing of a target gene in the plant genome or in the genome of the infecting pathogen, which is important for the regulated process or for the viability of the pathogen. There have been numerous studies where the utilization of exogenous dsRNA for virulence gene silencing in plant fungal pathogens has been documented [4,11,13], as well as studies on dsRNA antiviral effects [4,14,38]. However, there are only a few studies that have explored the effective silencing of plant genes through plant surface treatments with dsRNAs. Some studies and a patent have reported on the downregulation of plant genes through the external surface treatments with dsRNAs/siRNAs [17,18,19,20,21,22,39]. According to the studies, treating plants with dsRNAs encoding plant endogenous targets can downregulate mRNA levels of these genes. For instance, in tobacco and amaranth leaves, the transcript levels of the *EPSPS* gene were suppressed [39] and in orchid flower buds, the *Myb1* gene was silenced [17]. Similarly, treating grapevine with dsRNAs specific to the LBDIf7 [21] and GST40 [22] genes resulted in their downregulation. These studies have shown that exogenous plant treatments with target-specific dsRNAs can lead to desirable changes in plant phenotype or biochemistry, such as altered flower morphology, fungal resistance, and enhanced drought stress tolerance. In addition, the nanoparticle-mediated delivery of dsRNAs [24,26] and laser light-accompanied exogenous dsRNA treatments [25] have also been explored as a means to achieve plant gene silencing. However, exploring the efficient silencing of plant genes through surface treatments with dsRNAs is a relatively new area of research. Further research is needed to develop efficient approaches to inducing RNAi and specifically downregulating target genes in plants. 

In this investigation, the implications of applying five different dsRNAs, both individually and simultaneously, to the leaves of *A. thaliana* were examined. The goal was to suppress the expression of five specific genes that play a role in blocking or competing with the biosynthesis of anthocyanins, thereby influencing anthocyanin accumulation in the leaves of *A. thaliana*. Although anthocyanins can be found in various plant species, they are often found in limited quantities or are nonexistent in numerous plants as a result of the restricted activity of the flavonoid biosynthetic pathway [40,41].

The process of anthocyanin biosynthesis in Arabidopsis and other plants is regulated by the coordinated action of various transcription factors. These transcription factors work together to form a protein complex called the MYB–bHLH–WD repeat (MBW) complex. This complex consists of basic helix–loop–helix (bHLH), R2R3-MYB, and WD40-repeat proteins [42]. However, there are also specific single repeat R3-MYB transcription factors that negatively regulate anthocyanin biosynthesis. These include TRIPTYCHON (TRY), MYBL2, CAPRICE (CPC), ENHANCER OF TRY AND CPC 1 (ETC1), and ETC2, which interfere with the formation of the MBW protein complex, thereby inhibiting anthocyanin accumulation [30,42]. Furthermore, other molecular players such as ubiquitin protein ligases or additional transcription factors also contribute to the regulation of anthocyanin biosynthesis in Arabidopsis and other plants. For instance, a recent discovery identified the NAC transcription factor ANAC032 as a suppressor of anthocyanin biosynthesis in *A. thaliana* [31]. In our study, we focused on four negative regulators of anthocyanin biosynthesis (*AtANAC032*, *AtCPC*, *AtMYBL2*, *AtCBP60g*) and a competing enzyme *AtBAN*, and we demonstrated that the applied individual dsRNAs downregulated their expression while simultaneously increasing the expression of *AtCHS*.

By applying dsRNAs encoding the five regulators of anthocyanin accumulation to the leaves of *A. thaliana* plants, we observed a decrease in the expression of the five targets, while there was a simultaneous elevation in the mRNA levels of anthocyanin synthesis genes. This resulted in a significant elevation in the anthocyanin levels, as confirmed through HPLC-MS analysis. This study demonstrated that *A. thaliana* leaves contained a total of 0.01 to 0.02 mg/g FW anthocyanins when the five dsRNAs were separately applied under control conditions. However, when dsRNAs were applied simultaneously, the anthocyanin levels reached between 0.05 and 0.07 mg/g FW under control conditions. The simultaneous foliar application of all five dsRNAs proved to be more effective in promoting anthocyanin accumulation compared to individual dsRNA treatments. Overall, under anthocyanin-modulating conditions, the application of the exogenous dsRNAs in our study successfully boosted the anthocyanin levels in *A. thaliana* leaves to 0.29 mg/g FW, representing a significant increase. Thus, the efficiency of the exogenously induced RNAi appears to be stronger under the anthocyanin-inducing conditions, presumably due to activated anthocyanin biosynthesis, where the overall negative regulation of anthocyanin accumulation is restrained. While the exogenous dsRNAs reduced the expression of the five genes of anthocyanin negative regulators, there were a larger number of blockers that continued to restrain the biosynthesis of anthocyanins under control conditions at 22 °C. However, under the conditions stimulating the biosynthesis of anthocyanins (+7 °C, 23 h light), the effect of other blockers decreased, and the RNAi effects were modulated.

Several research studies have presented evidence supporting the involvement of AtCPC [29], AtMYBL2 [30], and AtANAC032 [31] transcriptional factors in exerting inhibitory effects on the production of anthocyanins in *A. thaliana*. In addition, several reports indicate that the calmodulin-binding protein AtCBP60g has a role in plant defense, drought tolerance, and abscisic acid sensitivity [43,44]. Recently, AtCBP60g has been found to repress levels of anthocyanins induced by kinetin, sucrose, or drought [33], while the *AtBAN* gene acts as an anthocyanin reductase that converts anthocyanidins to 2,3-*cis*-flavan-3-ols through a competing pathway [34]. Our findings confirm that silencing of *AtANAC032*, *AtBAN*, *AtCBP60g*, *AtCPC*, and *AtMYBL2* genes, which are all involved in anthocyanin biosynthesis repression or competition, leads to the activation of anthocyanin biosynthesis in *A. thaliana.* Thus, this study showed that these factors act as negative regulators of anthocyanin biosynthesis or suppress the biosynthesis of anthocyanins due to activity in a close competing biosynthetic pathway. Importantly, the application of the nonspecific *NPTII*-dsRNAs to *A. thaliana* had no impact on the production of anthocyanins or the expression of the *AtCHS* gene in the plant. This illustrates that the observed dsRNA-induced silencing effect on anthocyanin biosynthesis-related genes was specific to the sequence and not due to the application of the dsRNA itself.

In conclusion, these results shed new light on the exogenous plant foliar treatments utilizing a combination of gene-specific dsRNAs in terms of future applications. This innovative approach can be considered as a rapid and effective tool for silencing plant genes, enabling a comprehensive investigation of plant gene function. Moreover, it holds great promise for inducing the yield of valuable secondary compounds in the field of plant biotechnology.

## 4. Methods and Materials

### 4.1. Plant Materials 

To ensure sterility, the wild-type *A. thaliana* (cv. Columbia) seeds were subjected to vapor-phase sterilization according to a described procedure [45]. Subsequently, the sterized seeds were plated on solid ½ Murashige and Skoog (MS) medium for two days at a temperature of 4 °C. Then, the plates were transferred to a growth chamber (Sanyo MLR-352, Panasonic, Osaka, Japan) set at a light intensity of approximately 120 μmol m^−2^s^−1^ for a daily light period of 16 h at 22 °C. After one week, the one-week-old *A. thaliana* seedlings were transplanted into pots measuring 7 cm × 7 cm and filled with 100 g of nutrient-rich soil (the soil was irrigated using filtered water applied at the bottom of each pot). Thereafter, the plants were cultivated in the growth chamber, covered with a plastic wrap, for additional three weeks without further irrigation at 22 °C. Subsequently, the dsRNA treatments were applied exogenously to the four-week-old *A. thaliana* plants. Following the dsRNA application, the plants were placed in a growth chamber (KS-200, Smolenskoye SKTB SPU, Smolensk, Russia) and incubated for an additional seven days under either control conditions (22 °C, 16 h daily light period) or anthocyanin-inducing conditions (7 °C, 23 h daily light period), without further irrigation. This was performed to trigger the expression of *AtCHS* and subsequent accumulation of anthocyanins.

### 4.2. Isolation, Cloning, and Sequencing of *AtANAC032*, *AtCPC*, *AtCBP60g*, *AtMYBL2*, *and AtBAN* Genes

Full-length coding cDNA sequences of *AtANAC032 (AT1G77450*, 762 bp), *AtCPC (AT2G46410*, 285 bp), AtCBP60g (AT5G26920, *1080 bp)*, AtMYBL2 (AT1G71030.1, 588 bp), and *AtBAN (AF092912*, 1029 bp) genes were amplified through RT-PCR using RNA samples extracted from the adult leaves of *A. thaliana*. The RT-PCRs were carried out in a Bis-M1105 Thermal Cycler (Bis-N, Novosibirsk, Russia) with the primers listed in Table S1. Subsequently, the RT-PCR products were subcloned into pJET1.2/blunt and subjected to sequencing following a previously described protocol [46]. 

### 4.3. Design and Synthesis of dsRNAs

In order to produce the dsRNAs, we utilized the T7 RiboMAX™ Express RNAi System (Promega, Madison, WI, USA) to amplify the cloned full-length cDNAs of *AtCPC*, *AtMYBL2*, and *AtANAC032* as well as the large cDNA fragments of *AtCBP60g* (724 bp out of 1080 bp) and *AtBAN* (486 bp out of 1029 bp) via PCR. Additionally, we amplified a large fragment of *NPTII* (GenBank AJ414108, 599 bp out of 798 bp) using pZP-RCS2-nptII plasmid [47]. To achieve this, the T7 promoter sequence was introduced into both the 5′ and 3′ ends of the amplified *AtCPC*, AtCBP60g, AtBAN, AtMYBL2, AtANAC032, and *NPTII* in a PCR for each gene using primers listed in Table S1. The PCRs were conducted in the Bis-M1105 Thermal Cycler following the instructions of the T7 RiboMAX™ Express RNAi System. Subsequently, the obtained PCR products were employed as templates for in vitro transcription and dsRNA synthesis, in accordance with the manufacturer’s protocol. After in vitro transcription, the dsRNAs were treated with DNAse and RNAse and reprecipitated with ethanol according to the manufacturer’s recommendations (Promega, Madison, WI, USA). We assessed the resulting dsRNAs using gel electrophoresis (Figure S2) and spectrophotometry to verify dsRNA purity, integrity, and amount.

### 4.4. Application of Exogenous dsRNAs

To apply the dsRNAs, including the *AtCPC*, AtCBP60g, AtBAN, AtMYBL2, AtANAC032, and *NPTII*-dsRNAs, to individual four-week-old rosettes of wild-type *A. thaliana*, we used sterilized individual soft brushes (made of natural pony hair) [34]. For a separate application of dsRNA, we diluted 35 µg of the dsRNA in 100 µL of nuclease-free water for each treated rosette and spread it on the foliar surface using the brushes. Both the adaxial (upper) and abaxial (lower) sides of all leaves of one rosette were treated for each type of dsRNA. In the case of mixture application, we diluted equal quantities of each dsRNA (10, 20, or 30 µg of each dsRNA or 50, 100, and 150 mg total dsRNA in mixtures, respectively) in 100 µL of nuclease-free water and spread them on the foliar surface of each treated rosette. This resulted in a final concentration of 0.1, 0.2, or 0.3 µg/µL of each dsRNA, equivalent to 0.5, 1.0, or 1.5 µg/µL of total dsRNAs. Both the adaxial and abaxial sides of all leaves of one rosette were treated for each type of mixture. In each independent experiment, one plant of *A. thaliana* was treated with 100 µL of the dsRNA of each type, while another plant received 100 µL of sterile filtered water. It should be noted that the dsRNAs were applied to four-week-old rosettes of *A. thaliana* late in the day (21:00–21:30) under low soil moisture conditions. This was based on our recent analysis, which determined these conditions as crucial parameters for successful target gene suppression in *A. thaliana* [34]. The soil water content before dsRNA treatment was 50–60%.

### 4.5. RNA Isolation and Reverse Transcription

To isolate total RNA, we collected a typical adult L3 leaf [48] from a single *A. thaliana* plant before any treatment. Additionally, we collected leaves two days and seven days after each specific treatment. The cetyltrimethylammonium bromide (CTAB)-based protocol [49] was employed to extract total RNA. Subsequently, complementary DNAs were synthesized as previously described [50].

### 4.6. Analysis of Gene Expression Using qRT-PCR

The PCR-amplified reverse transcription products were examined for the absence of DNA contamination using primers listed in Table S1. qRT-PCR was performed using SYBR Green I Real-time PCR dye and a real-time PCR kit (Evrogen, Moscow, Russia), following the methodology described by [51]. Two internal controls, GAPDH and UBQ, which were previously validated as suitable reference genes for qRT-PCR analysis in Arabidopsis [52], were included. The expression levels were determined using the 2^−ΔΔCT^ method [53]. The obtained data for target gene expression at seven and two days post treatment were divided to the expression levels of the target gene before treatment, indicating fold change in target gene expression relative to the respective data before treatment. The gene identification numbers and the primers used in this study are listed in Table S1.

### 4.7. HPLC-MS Analysis of Anthocyanins

The frozen *A. thaliana* rosettes that underwent treatment were homogenized using a mortar and pestle. Shredded tissue was weighed and then subjected to extraction in 1 mL of 1% (*v*/*v*) hydrochloric acid and methanol for 24 h at 4 °C. Afterwards, the mixture was centrifuged at 13,200 rpm for 15 min. To prepare samples for HPLC–MS analysis, a 0.45-um nylon filter was used for sample filtration. All anthocyanins present were identified using an Agilent Technologies 1260 Infinity analytical HPLC system (Santa Clara, CA, USA) coupled with Bruker HCT ultra PTM Discovery System (Bruker Daltonik GmbH, Bremen, Germany) that featured an electrospray ionization (ESI) source, according to a protocol described previously [20]. A Shimadzu HPLC LC-20AD XR analytical system (Kyoto, Japan) equipped with diode array detection (HPLC–DAD) was employed for quantification of all anthocyanins following previously described procedures [20]. The anthocyanin contents were determined using an external standard method with the four-point regression calibration curves constructed using available standards. As a control, the commercial standard cyanidin chloride, obtained from Sigma-Aldrich (St. Louis, MO, USA), was used.

### 4.8. Statistical Analyses

The data are presented as mean ± standard error (SE) and were tested through a paired Student’s *t*-test. The level of *p* < 0.05 was chosen as the point of minimal statistical significance in all analyses. Each type of analysis was repeated in a minimum of three independent experiments.

## Data Availability

The data presented in this study are available within the article and Supplementary Materials.

## Supplementary Materials

The following supporting information can be downloaded at: https://www.mdpi.com/article/10.3390/plants13040541/s1, Figure S1: HPLC chromatogram of anthocyanins in *Arabidopsis thaliana* leaves examined seven days after treatment with dsRNA mixture of five dsRNAs of *AtANAC032*, *AtCBP60g*, *AtCPC*, *AtMYBL2*, and *AtBAN* gene under anthocyanin-induction conditions (A. 150 mg total dsRNA, +7 °C, 23 h light) or with water (B. H_2_O); Figure S2: Electrophoretic separation of used dsRNAs in 2% agarose gel. *ANAC032-dsRNA* (a), *AtCBP60g*-dsRNA (b), *AtCPC*-dsRNA (c), *AtMYBL2*-dsRNA (d), *AtBAN*-dsRNA (e), and *NPTII*-dsRNA (f); Table S1: Primers used in RT-PCR and qRT-PCRs; Table S2: List of anthocyanins identified in the methanol extracts of *Arabidopsis thaliana* rosettes.
