# Supplementary material for: Simultaneous Application of Several Exogenous dsRNAs for the Regulation of Anthocyanin Biosynthesis in Arabidopsis thaliana
Source: Plants (Basel). 2024 Feb 16;13(4):541. doi: 10.3390/plants13040541 (PMC10893326; doi:10.3390/plants13040541)
Supplement: Supplementary file 1 [file plants-13-00541-s001.zip › plants-2824415-supplementary.pdf]

Supplementary Table S1. Primers used in RT-PCR and qRT-PCRs.

Gene name (ID number)	Primer name	Primers, 5′-3′
Primers for cloning full-length cDNA coding sequences, 5′-3′		
AtANAC032 (AT1G77450.1)	ANAC032-s1, ANAC032-kon	5′ATGATGAAATCTGGGGCTG, 5′TCAGAAAGTTCCCTGCCTA
AtCBP60g (AT5G26920.1)	CBP60g-s1, CBP60g-kon	5′ATGAAGATTCGGAACAGCCC, 5′TTACAAGCCTTCCCTCGGAT
AtCPC (AT2G46410.1)	CPC-s1, CPC-kon	5′ATGTTTCGTTCAGACAAGGC, 5′TCATTTCCTAAAAAAGTCTC
AtMybL2 (AT1G71030.1)	AtMybL2-s1, AtMybL2-kon	5′ATGAACAAAACCCGCCTTC, 5′TCATCGGAATAGAAGAAGC
AtBAN (NM_104854.4)	BAN-s1, BAN-kon	5′ATGGACCAGACTCTTACACAC, 5′TTATTTAGCTTTGATCAATCC
Specific primers for dsRNA design, 5′-3′		
AtANAC032 (AT1G77450.1)	ANA-dsRNA-s1, ANA-dsRNA-a1	5'TAATACGACTCACTATAGGGAGAATGATGAAATCTGGGGCTGATTTG, 5'TAATACGACTCACTATAGGGAGATCAGAAAGTTCCCTGCCTAACC
AtCBP60g (AT5G26920.1)	CBP60g-dsRNA-s1, CBP60g- dsRNA-a1	5'TAATACGACTCACTATAGGGAGAATGAAGATTCGGAACAGCCC, 5'TAATACGACTCACTATAGGGAGACATCACTGGGGCACGGAGGATG
AtCPC (AT2G46410.1)	CPC-dsRNA-s1, CPC-dsRNA-a1	5'TAATACGACTCACTATAGGGAGACTTGTTCCGAAGAGGTGAGT, 5'TAATACGACTCACTATAGGGAGATCATTTCCTAAAAAAGTCTC
AtMybL2 (AT1G71030.1)	MybL2-dsRNA-s1, MybL2-dsRNA-a1	5'TAATACGACTCACTATAGGGAGAATGAACAAAACCCGCCTTC, 5'TAATACGACTCACTATAGGGAGATCATCGGAATAGAAGAAGCGTTT
AtBAN (NM_104854.4)	BAN-dsRNA-s1, BAN-dsRNA-a1	5'TAATACGACTCACTATAGGGAGAATGGACCAGACTCTTACACAC, 5'TAATACGACTCACTATAGGGAGACTCCTCTGTGAGAAATTCAACG
NPTII (AY818371)	npt-T71-s, npt-T72-a	5'TAATACGACTCACTATAGGGAGAATGTGGATTGAACAAGATGGATTG, 5'TAATACGACTCACTATAGGGAGATCCACCATGATATTCGGCAAGCAG
Primers for cDNA check-up on DNA contamination, 5′-3′		
AtGAPDH (GenBank NM_111283)	AtGapdh-s, AtGapdh-a	5′CTG GAA TGT CTT TCC GTG TC, 5′ATT CGT TGT CGT ACC ATG AC
Primers for PCR and real-time PCR, 5′-3′		
AtANAC032 (AT1G77450.1)	ANAC032-reals1, ANA-3UTR-real-a1	5′CGTTTAATTACGTAGATGC, 5′CACTTTCCACTAACTCTAATCGC

AtCBP60g (AT5G26920.1)	CBP60g-real-s1, CBP60g-real-a1	5′GGTGTAAGGTTAAGGCAGCTTTC, 5′TCTGGCAGTTGTGTGTCTCC
AtCPC (AT2G46410.1)	CPC-real-s1, CPC-real-a1	5′ACGACGGAGACAGAGCAAAG, 5′CTGACATCTTCACAGCTTCC
AtBAN (NM_104854.4)	BAN-reals1, BAN-realA1	5′CTCTCCTCTCCGATCCTCCG, 5′GCAAATGTAGCGACCAGAAGCAG
AtMybL2 (AT1G71030.1)	AtMybL2-reals1, MybL2-3UTR-realA1	5′TTGCCTGACCTAAACATTG, 5′GCCGGTCCAATTCAGGATTAAC
NPTII (GenBank AJ414108)	nptII-realS, nptII-realA	5′TTGCTGAAGAGCTTGGCGGCGAAT, 5′TCAGAAGAACTCGTCAAGAAGG
AtGAPDH (GenBank NM_111283)	AtGapdh-real-s, AtGapdh-real-a	5′TTG GTG ACA ACA GGT CAA GCA, 5′AAA CTT GTC GCT CAA TGC AAT
AtUBQ (GenBank NM_001084884)	AtUBQ-realS, AtUBQ-realA	5′GGCCTTGTATAATCCCTGATGAATAAG, 5′AAAGAGATAACAGGAACGGAAACATAGT

Supplementary Table S2. List of anthocyanins identified in the methanol extracts of *Arabidopsis thaliana* rosettes.

Peak	By Tohge et. al. 2005 [35]	Rt (min)[a]	UV, λmax (nm)	MS (m/z)	MS^2 fragments (m/z)	Name
1	A8	19,7	527	1137	535,889	Cyanidin 3-O-[2´´-O-(xylosyl) 6´´-O-(p-O-(glucosyl) p-coumaroyl) glucoside] 5-O-[6´´´-O-(malonyl) glucoside]
2	A10	20,8	520	1258	1095,449	Cyanidin 3-O-[2´´-O-(2¢¢¢-O-(sinapoyl) xylosyl) 6´´-O-(p-O-(glucosyl) p-coumaroyl) glucoside] 5-O-glucoside
3	A11a	21,8	540	1344	1095,535	Cyanidin 3-O-[2´´-O-(6´´´-O-(sinapoyl) xylosyl) 6´´-O-(p-O-(glucosyl)-p-coumaroyl) glucoside] 5-O-(6´´´´-O-malonyl) glucoside
4	A11b	22,8	535	1344*	1095,535	
5	A7	24,1	530	1095	933,449	Cyanidin 3-O-[2´´-O-(2´´´-O-(sinapoyl) xylosyl) 6´´-O-(p-coumaroyl) glucoside] 5-O-glucoside
6	A5	25	525	975	727,535,287	Cyanidin 3-O-[2´´-O-(xylosyl)-6´´-O-(p-coumaroyl) glucoside] 5-O-malonylglucoside
7	A9a	26	530	1182	1137,933,535,491	Cyanidin 3-O-[2´´-O-(2´´´-O-(sinapoyl) xylosyl) 6´´-O-(p-O-coumaroyl) glucoside] 5-O-[6´´´´-O-(malonyl) glucoside]
8	A9b	26,8	535	1182*	1137,933,535,491	

*Asterisk indicates a tautomer.

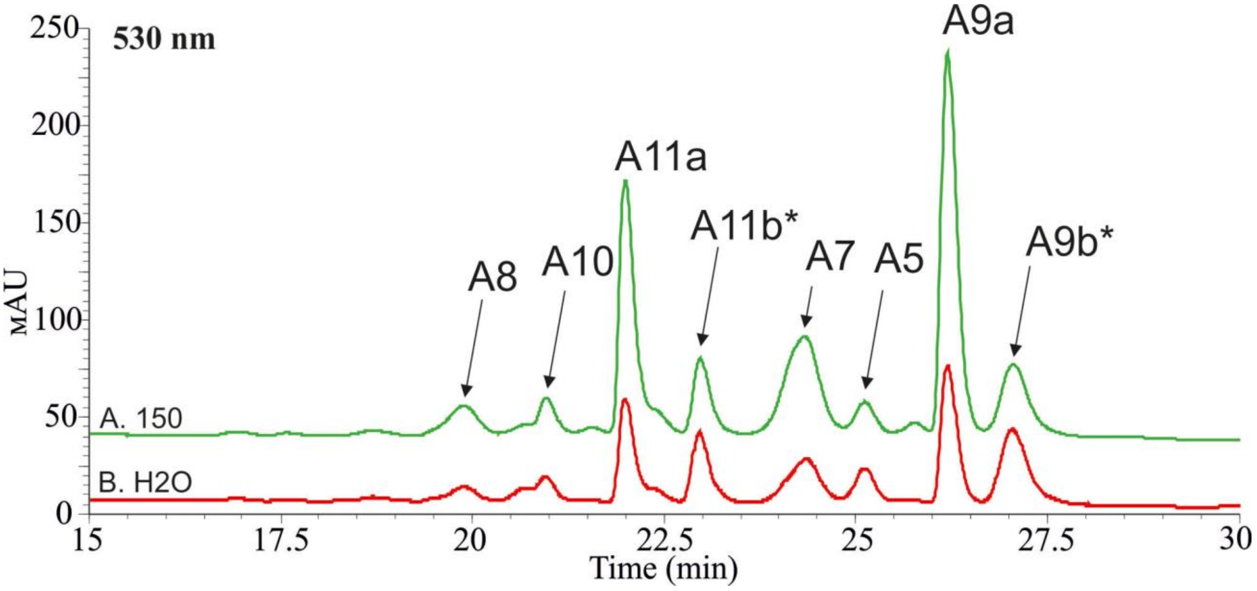

Supplementary Figure S1. HPLC chromatogram of anthocyanins in *Arabidopsis thaliana* leaves examined seven days after treatment with dsRNA mixture of five dsRNAs of *AtANAC032*, *AtCBP60g*, *AtCPC*, *AtMYBL2*, and *AtBAN* gene under anthocyanin-induction conditions (A. 150 mg total dsRNA, +7°C, 23 h light) or with water (B. H2O).

A8 - Cyanidin 3-*O*-[2′′-*O*-(xylosyl) 6′′-*O*-(*p*-O-(glucosyl) p-coumaroyl) glucoside] 5-*O*-[6′′′-*O*-(malonyl) glucoside]; A10 - Cyanidin 3-*O*-[2′′-*O*-(2′′′-*O*-(sinapoyl) xylosyl) 6′′-*O*-(*p*-*O*-(glucosyl) *p*-coumaroyl) glucoside] 5-*O*-glucoside; A11a, A11b* - Cyanidin 3-*O*-[2′′-*O*-(6′′′-*O*-(sinapoyl) xylosyl) 6′′-*O*-(*p*-*O*-(glucosyl)-p-coumaroyl) glucoside] 5-*O*-(6′′′′-*O*-malonyl) glucoside; A7 - Cyanidin 3-*O*-[2′′-*O*-(2′′′-*O*-(sinapoyl) xylosyl) 6′′-*O*-(*p*-coumaroyl) glucoside] 5-*O*-glucoside; A5 - Cyanidin 3-*O*-[2′′-*O*-(xylosyl)-6′′-*O*-(p-coumaroyl) glucoside] 5-*O*-malonylglucoside; A9a, A9b*- Cyanidin 3-*O*-[2′′-*O*-(2′′′-*O*-(sinapoyl) xylosyl) 6′′-*O*-(*p*-*O*-coumaroyl) glucoside] 5-*O*-[6′′′′-*O*-(malonyl) glucoside]. *Asterisk indicates a tautomer. The names of the anthocyanins are presented in accordance with previously published data [35].

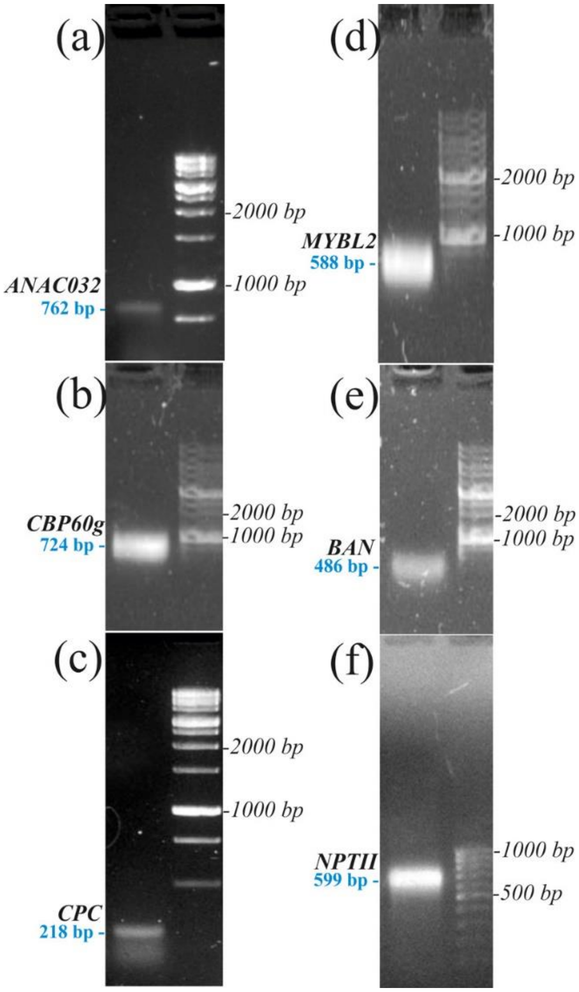

Supplementary Figure S2. Electrophoretic separation of used dsRNAs in 2% agarose gel. *ANAC032*-dsRNA (a), *AtCBP60g*-dsRNA (b), *AtCPC*-dsRNA (c), *AtMYBL2*-dsRNA (d), *AtBAN*-dsRNA (e), and *NPTII*-dsRNA (f).
